# Effect of high time under tension strength training on different muscular actions in the performance of runners: A randomized controlled trial

**DOI:** 10.1371/journal.pone.0342428

**Published:** 2026-02-09

**Authors:** Júlio César de Carvalho Martins, Matheus Santos de Sousa Fernandes, Felipe J. Aidar, Gustavo Ivo de Carvalho e Silva, Eder Magnus Almeida Alves Filho, Fatma Hilal Yagin, Georgian Badicu, Pablo Prieto González, Sameer Badri Al-Mhanna, Mohammed Dauda Goni, Nouf H. Alkhamees, Raphael Fabricio de Souza

**Affiliations:** 1 Postgraduate Program of Movement Science, Federal University of Sergipe, São Cristóvão, Brazil; 2 Keizo Asami Institute, Federal University of Pernambuco, Recife, Pernambuco, Brazil; 3 Department of Biostatistics, Faculty of Medicine, Malatya Turgut Ozal University, Malatya, Turkey; 4 Department of Computer Science, Lakehead University, Thunder Bay, Ontario, Canada; 5 Department of Physical Education and Special Motricity, Faculty of Physical Education and Mountain Sports, Transilvania University of Braşov, Braşov, Romania; 6 Sport Sciences and Diagnostics Research Group, GSD-HPE Department, Prince Sultan University, Riyadh, Saudi Arabia; 7 Department of Physiology, Saveetha Medical College and Hospitals, Saveetha Institute of Medical and Technical Sciences, Saveetha University, Chennai, India; 8 Department of Higher Studies, Al-Qasim Green University, Babylon, Iraq; 9 Faculty of Veterinary Medicine, Universiti Malaysia Kelantan, Kelantan, Malaysia; 10 Department of Rehabilitation Sciences, College of Health and Rehabilitation Sciences, Princess Nourah bint Abdulrahman University, Riyadh, Saudi Arabia; Università degli Studi di Milano: Universita degli Studi di Milano, ITALY

## Abstract

**Background:**

Strength training (ST) for runners is typically based on low volumes and high intensities. However, alternative approaches emphasizing higher volume and lower intensity, such as protocols with high time under tension (TUT), remain underexplored in this population. Notably, the effects of high-TUT may vary depending on the predominant type of muscle contraction.

**Objectives:**

To analyze the impact of strength training with high time under tension on different muscle actions in performance-related variables in runners.

**Methods:**

Thirty-four physically active young males were randomly divided into three groups: dynamic strength training, isometric strength training, and a control group. Over four weeks, the training groups performed two weekly sessions of bodyweight exercises with equalized time under tension per set (84 s), adjusted according to the type of contraction. The primary outcomes were performance in a 3000-meter time trial, peak torque, countermovement jump, neuromuscular fatigue, internal running load, ground contact time, and vertical oscillation. The data were analyzed using a two-way repeated measures analysis of variance.

**Results:**

Peak torque increased by 13.3% in the dynamic group and 14.2% in the isometric group, compared with 2.5% in the control, with statistical significance only for the isometric group (*p* = 0.034, *d* = 1.12). Vertical jump height improved by 5.4% in the dynamic group and 4.1% in the isometric group compared with 0.7% in the control (*p* = 0.003, *d* = 1.54 and *p* = 0.030, *d* = 1.13, respectively).

**Conclusion:**

In conclusion, high time under tension strength training, both dynamic and isometric, improved neuromuscular characteristics in runners. However, these adaptations did not translate into significant changes in running performance or running economy over the duration of the intervention.

**Trial registration:**

This trial was registered in the Brazilian Clinical Trials Registry (ReBEC) with the identifier RBR-686kqdx.

## Introduction

In middle- and long-distance running, although performance predominantly depends on aerobic capacity and the ability to sustain prolonged efforts [1], anaerobic and neuromuscular characteristics also exert significant influence, primarily through running economy (RE), which can be improved with strength training (ST) [2]. RE is also strongly influenced by biomechanical factors related to how force is generated and applied during running. Studies have shown that variables such as ground contact time, lower-limb stiffness, and mechanical work explain substantial portions of the inter-individual variability in RE and predict long-distance performance in runners [3,4]. Additionally, running technique, particularly the efficiency of force transfer to the ground, has been identified as a key determinant of both RE and performance [5]. Together, these findings indicate that neuromuscular capacity and biomechanical efficiency play a central role in running performance, providing a theoretical basis for interventions aimed at enhancing these determinants.

A recent review indicated that ST enhances intra- and intermuscular coordination, the stretch-shortening cycle (SSC), the rate of force development (RFD), and the strength of both type I and type II muscle fibers in runners [6]. These adaptations reduce motor unit recruitment and optimize force application during running, improving biomechanical efficiency and reducing the physiological demand to maintain a given speed.

Such adaptations have been mainly observed across various ST methods, including maximal and submaximal loads, plyometrics, and combined training, as well as training variables such as volume, intensity, and contraction speed [6]. However, one variable that has been only partially explored in the literature on running performance is time under tension (TUT), which refers to the duration of muscular effort in a set. It is categorized as low (9–15 s) or high-TUT (60–105 s), with the magnitude of effort depending on the contraction speed of each repetition [1,7].

Traditionally, stimuli involving low TUT and fast contractions, such as submaximal loads and plyometrics, are beneficial for neuromuscular function and RE [6,8], can induce greater mechanical stress, potentially leading to increased muscle damage [9], reduced maximal strength, and impaired jump capacity compared to slower contractions [10], thereby hindering recovery. On the other hand, protocols with high-TUT and slow contractions induce greater metabolic stress via intramuscular hypoxia, acutely stimulating mitochondrial protein synthesis [1,11]. However, it is known that in ST protocols with higher volume and lower intensity, peripheral aerobic adaptations tend to follow neuromuscular improvements in untrained individuals [1,12]. This suggests that high-TUT may induce short-term neuromuscular adaptations in runners and enhance performance, although this hypothesis has yet to be tested.

The impact of this strategy, however, may vary according to the type of muscular action, mainly because they operate through distinct mechanisms [13]. Dynamic actions (concentric and eccentric), usually performed with fast contractions, have shown improvements in time trial performance and vertical jump ability [6,14]. Isometric actions, typically executed with slow contractions, have demonstrated positive effects on time trial performance, maximal strength, and dynamic parameters such as reduced ground contact time [14,15]. Although both dynamic and isometric actions appear to promote neuromuscular adaptations, methodological variability across studies, especially concerning TUT control and execution speed, hinders direct comparisons and limits our understanding of the effects of high-TUT in runners. Thus, equalizing high-TUT and slow pace across different muscle action types may represent a promising strategy to enhance performance in runners.

Therefore, the objective of this study was to analyze the effect of ST with high-TUT using predominantly dynamic and isometric actions on performance-related variables in runners. We hypothesize that high-TUT, regardless of contraction type, improves running performance indicators, neuromuscular strength, and biomechanical variables related to RE. Furthermore, this type of training is expected to modulate neuromuscular fatigue without inducing high levels after exhaustive effort.

## Materials and methods

### Research ethics

After being fully informed about the study’s objectives and procedures, all participants signed the Informed Consent Form. This study followed the ethical principles outlined in the Declaration of Helsinki of 1964 (revised in October 2013). It was approved by the Human Research Ethics Committee of the Federal University of Sergipe (6.997.352) and registered in the Brazilian Registry of Clinical Trials (ReBEC), available at https://ensaiosclinicos.gov.br/rg/RBR-686kqdx, with a registration date of August 14, 2024.

### Participants

Forty physically active young male runners, all serving as Brazilian Army soldiers, were recruited for the study. The participants were previously trained in endurance, maintaining a weekly running routine of 12–22 km both inside and outside the barracks. Additionally, they participated in one weekly session of operational cross-training, which included strength exercises and other physical capacities essential for military tasks. Thus, the runners were not highly trained in strength. The trial was conducted at the Tiro de Guerra 06–015 (TG 06–015), located in Lagarto, Sergipe, Brazil.

Inclusion criteria were: 1) being ≥ 18 years old; 2) running at least three times per week with an average weekly mileage ≥ 12 km; 3) having an average running pace between 4:00 and 5:30 min/km in long-distance events (3000 m); 4) participating in a strength training program at least once a week for a minimum of three months; 5) reporting no musculoskeletal injuries in the previous three months; 6) having at least six months of experience in long-distance running (3000 meters); and 7) attending at least 87.5% of the training sessions. Exclusion criteria were: 1) performing physical exercise and/or consuming alcohol 24 hours before tests or training; and 2) consuming caffeine within 4 hours before tests or training. Before training sessions, participants were questioned about their activities on the previous day.

Similar to the approach proposed by Li et al. [16], he matching process was performed entirely using Python (version 3.12.5). For this purpose, equation (1) of the Euclidean Distance $\left(ED=\sqrt{\left(x1-x2\right)2+\left(y1-y2\right)2}\right)$ was used. First, the script automatically calculated the Euclidean Distance between all possible pairs of participants based on the raw values of age and weekly running mileage, variables directly related to running performance. These distances were then used in a hierarchical clustering algorithm, implemented in Python, which grouped participants into triplets containing the most similar individuals. After the formation of these triplets, randomization was also performed automatically by the script using a random number generator (Python’s random module, with a fixed seed to ensure reproducibility). Within each triplet, one participant was assigned to each of the three experimental groups, ensuring balance across the characteristics used for matching.

The entire procedure, distance calculation, cluster formation, and randomization, was automated in Python, ensuring allocation concealment until the participants were assigned to their respective groups.

Only the participants were blinded to group allocation, while the researchers responsible for training supervision and outcome assessments were aware of the group assignments. Blinding of participants was achieved by providing limited information about the specific characteristics of each training protocol, so that participants were unaware of the group to which they were assigned. Although the interventions differed in contraction type (dynamic vs. isometric), sessions were conducted in a similar environment and format to minimize recognition of group allocation.

**Fig 1:**
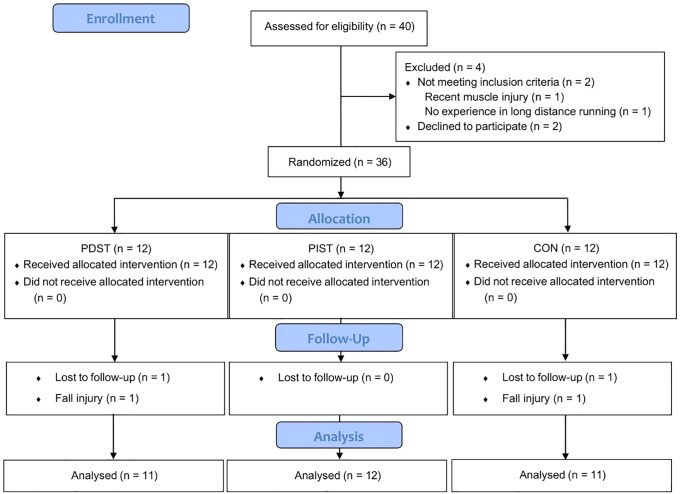
Flow diagram of the study participants.

Of the forty recruited runners, four were excluded after the eligibility process due to not meeting the inclusion criteria (n = 2) and withdrawal from the study (n = 2). Thus, 36 participants started the study, equally distributed among the groups: PDST (predominantly dynamic strength training; n = 12), PIST (predominantly isometric strength training; n = 12), and CON (control; n = 12). During the study, one individual from the PDST group and one from the PIST group were excluded after sustaining injuries from falls unrelated to the study activities. Therefore, the final sample consisted of 34 runners, distributed as follows: PDST (n = 11), PIST (n = 12), and CON (n = 11). Fig 1 shows the flow diagram of the participants.

### Study design

This six-week randomized controlled trial was conducted in accordance with the CONSORT 2025 guidelines for randomized controlled trials (accessible in S1 File – Checklist) [17]. For four weeks, runners in the PDST and PIST groups performed bodyweight strength training with high-TUT per set, emphasizing concentric/eccentric and isometric actions, respectively.

**Fig 2:**
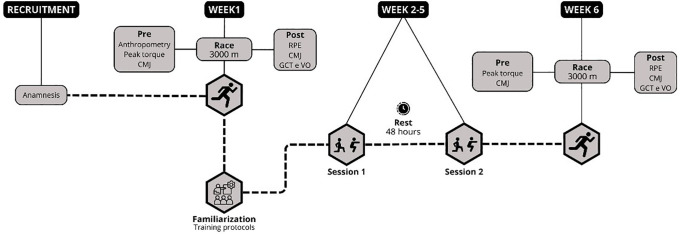
Study design. CMJ = counter movement jump, GCT = ground contact time, VO = vertical oscillation, RPE = rating of perceived exertion.

Participant recruitment was conducted from 30/08/2024 to 02/10/2024, during which personal information, including age and weekly running mileage, was collected from all participants. The study design, illustrated in Fig 2, included assessments conducted before and after the intervention, with measurements taken 2–3 minutes before the 3000 m time trial and immediately after its completion. Participants were also familiarized with the training protocols before the start of the intervention. Post-intervention assessments were conducted between 72 and 96 hours after the final training session.

To avoid variations caused by the circadian cycle, the pre-intervention, intervention, and post-intervention sessions were conducted at the same time of day, previously scheduled with each participant. All information regarding adverse effects was collected based on participants’ reports during training sessions. In the event of a report related to a serious injury, the event was recorded, and the individual was immediately withdrawn from the study to ensure participant safety.

The participants in this study were not involved in the design, conduct, or reporting of the trial. Study protocol details are available in PDF format with this article (accessible in S2 File – Study protocol).

### Sample size calculation

The sample size was calculated a priori based on the study by Lum et al. [14], which reported a partial eta-squared (*η²p*) effect size of 0.47 in the analysis of different strength training interventions on 2400-meter running performance in distance runners. G*Power software (version 3.1.9.7, University of Kiel, Kiel, Germany) was used, setting α < 0.05 and β = 0.80 in the statistical configuration of repeated-measures two-way ANOVA tests. The analysis revealed that 36 participants would be sufficient to observe a group (PDST vs. PIST vs. CON) × time (pre vs. post) interaction with a statistical power of 0.80.

### Training protocols

For four weeks, the training protocols were applied twice a week, with a 48-hour interval between sessions, resulting in a total of eight sessions by the end of the intervention [18]. The protocols focused on the lower limbs and included the following exercises: squat, unilateral lunge, supine hip lift, and plantar flexion. These exercises were selected based on studies demonstrating benefits for running performance [6,19].

Four sets per exercise were adopted, based on the average number of sets used in studies with runners, which range from 3 to 5 sets [6]. Three factors determined the intensity of the bodyweight exercises: 1) the low adherence of runners to strength training [20]; 2) the high metabolic stress generated by the slow pace of the exercises [1], which increased the training volume; and 3) the positive results shown by bodyweight training protocols [6]. Additionally, progressive overload was not included due to the sample’s low experience with strength training and the short intervention period.

To determine the ideal TUT per set for the sample, pilot tests were conducted using TUTs of 60, 84, and 105 seconds. It was observed that 60 seconds and 105 seconds produced, respectively, low and high metabolic stress stimuli. Therefore, a TUT of 84 seconds was chosen as it represented a balance between neuromuscular stimulation and fatigue control. Moreover, two sets per leg were performed for the unilateral lunge due to the high fatigue perception reported by participants in the final repetitions when performing four sets, which compromised execution.

Regarding the TUT per repetition, the protocols were based on evidence demonstrating positive effects on strength. The PDST followed a 3/1/3/0 cadence (3 seconds concentric, 1 second isometric, and 3 seconds eccentric), based on Tanimoto and Ishii [21] and the recommendations of Davies et al. [22] and Mang et al. [1]. The PIST used a 1/5/1/0 cadence (1 second eccentric, 5 seconds isometric, and 1 second concentric), adapted from Gentil et al. [1], and also suggested by Mang et al. [1]. Both protocols were equalized in terms of the number of exercises, sets, rest intervals, and total session time. To achieve the 84 seconds of TUT per set, 12 repetitions were prescribed, totaling 7 seconds per repetition for both protocols Mang et al. [1]. Before each session, a specific warm-up was performed according to the characteristics of each protocol [19,23]. The CON group did not undergo any training and was instructed to maintain their usual daily activities throughout the intervention period.

**Fig 3:**
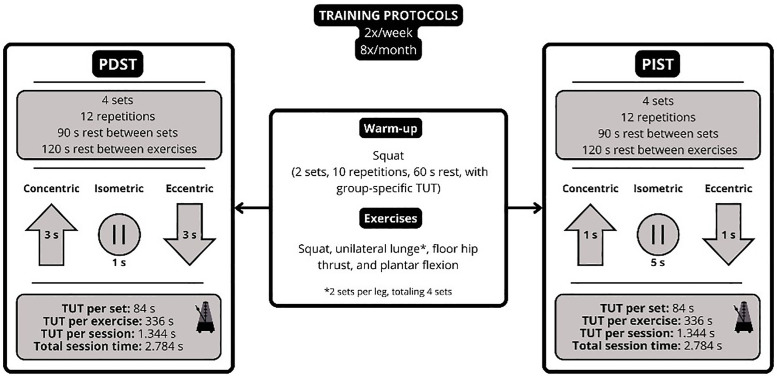
Design of training protocols. PDST = predominantly dynamic strength training, PIST = predominantly isometric strength training, TUT = time under tension.

A 90-second rest interval was adopted between sets. Between exercises, the recovery period was 120 seconds. TUT was also strictly monitored using a metronome, and all sessions were conducted in groups. Fig 3 summarizes the structure of the protocols.

The participants were instructed to maintain their regular running training and weekly mileage as usual during the intervention period, except on the designated days for the experimental sessions. Weekly running volume was monitored through self-report. In all strength training sessions, a specialized Physical Education professional was present to ensure the correct manipulation of TUT in each protocol. In addition, participants were asked not to engage in any other strength training programs beyond those proposed in this investigation and to maintain their eating habits and sleep patterns without modification, following a routine throughout the six weeks.

### Anthropometry

Body mass and height were measured using an analog scale with a stadiometer and an accuracy of 0.1 kg (Filizola, MIC 2B/A Mechanical Anthropometric 300 kg, Brazil). Participants were barefoot and wore light clothing to ensure measurement accuracy.

### 3000-meter time trial

The 3000-meter time-trial field test (TT) was used in this study because it represents an event compatible with the regular practice of runners. The TT was therefore conducted on a track at two time points (pre-intervention and post-intervention). Running time was recorded in both trials using a digital stopwatch (Vollo Sports, model VL515, São Paulo, Brazil) and a GPS watch (Garmin Ltd, model Forerunner 245, Kansas, United States) [24] to ensure greater accuracy. Participants were instructed correctly and familiarized with the “start” and “stop” functions of the watch.

The warm-up consisted of two exercises, based on the recommendations of Wei et al. [25] and Pieters et al. [26]: squat jumps and horizontal jumps (2 sets of 8 repetitions for both exercises, with 30 seconds of rest between sets). The ambient temperature on testing days ranged between 25°C and 28°C. Runners performed the test individually and were encouraged during the run with clapping and motivational words. At the end of the time trial, participants were not informed of the number of laps completed or their final time. The time-trial result was recorded in seconds.

Additionally, the study by Aandstad [8] demonstrated that the 3000-meter TT is a valid and reliable tool for estimating VO_₂max_. Thus, its use in the present study enabled not only a specific evaluation of running performance after the intervention but also an indirect estimate of participants’ aerobic capacity. This estimate was calculated using the average speed (AS) obtained in the pre-intervention test to characterize the sample. The following equations (2) were used for the estimate:


AS=distanceinmeters÷timeinseconds×3,6

VO2max=17.5+2.57×AS

### Subjective perception of effort and internal running load

The Borg CR-10 rating of perceived exertion (RPE) scale [27], adapted by Foster et al. [28]. RPE was assessed at two time points during the time trial: immediately after time trial 1 and immediately after time trial 2. Because maximal or near-maximal efforts commonly elicit saturated RPE values (i.e., values close to the ceiling of the scale), RPE alone may fail to reflect subtle physiological or perceptual adaptations across repeated maximal tests [29].

To address this limitation, we calculated the internal running load (IRL), adapted from Sant’Ana et al. [30], by multiplying the time to complete the 3000 m trial (in minutes) by the corresponding RPE value. This composite metric provides a more sensitive indicator of the internal response to effort, as it incorporates both subjective perception and actual performance duration, allowing us to identify whether any of the high-TUT protocols could modulate this response.

The following equation (3) was used for the calculation:


IRL=totaltimetrialtimeinminutes×RPE

### Peak torque

Peak torque (PT) was collected as the maximum isometric torque produced by the knee extensor muscles, considering its direct association with running biomechanics and movement symmetry [31]. PT was determined by multiplying the peak isometric force of the quadriceps (dominant leg) by the segment length, defined as the distance between the load cell attachment point and the central axis of the knee joint. Isometric force was measured using a load cell (Kratos, model CZC500, São Paulo, Brazil), which was attached to an inextensible rope and positioned near the malleolus using Velcro. Additionally, a 70-degree knee flexion angle was used during isometric testing, as measured with a digital goniometer. Before the test, runners performed three familiarization attempts with the device. Afterwards, they completed three maximal attempts, and the average value was used for analysis. Participants were instructed to produce torque quickly and forcefully for a short period (approximately 2–3 seconds). A one-minute rest interval was observed after each effort [32]. Test values were reported in Newton-meters (Nm).

### Countermovement jump

The countermovement jump (CMJ) assessment was performed based on its relationship with neuromuscular adaptations and the quantification of neuromuscular fatigue [33]. Thus, the CMJ was collected at pre- and post-intervention moments, before the time trials, to evaluate neuromuscular capacity through jump height [14]. Additionally, measurements were taken immediately after the runs to estimate the acute percentage variation of neuromuscular fatigue (NF) at both time points [33,34] using the following equation (4): (NF=(CMJpost−CMJpre)÷CMJpre×100). The NF values from the pre- and post-intervention periods were subsequently used to calculate the percentage change in NF between the intervention groups.

The tests were performed using a contact mat (Chronojump-Bosco System, Barcelona, Spain) connected to computer software. Participants were warmed up and familiarized with the task before performing the pre-run jumps. Regarding the jump procedure, each participant began in an upright position, with their feet fixed on the mat at shoulder width and hands placed on their hips. They then performed a downward movement by flexing their knees to approximately 90° to jump to their maximum capacity quickly. Participants were asked to perform three consecutive jumps with a 1-minute rest interval between attempts. For data analysis, the highest jump height in centimeters was considered [35].

### Ground contact time and vertical oscillation

Ground contact time (GCT) and vertical oscillation (VO) were collected using a Garmin HRM-Run accelerometer (Garmin Ltd, Kansas, United States) in conjunction with a Garmin Forerunner 245 watch (Garmin Ltd, Kansas, United States). GCT and VO refer, respectively, to the time each foot spends in contact with the ground and the vertical movement of the body during running. Both variables were collected using instruments that have demonstrated validity and reliability in detecting dynamic running parameters [36]. GCT was expressed in milliseconds (ms), and VO in centimeters (cm), based on the average values recorded during time trials 1 and 2.

Vertical oscillation was collected as a complementary outcome. This variable was not specified in advance in the trial registration at ReBEC. Still, it was obtained because the wearable device used (Garmin HRM-Run) provided this data automatically during running performance tests.

### Statistical analysis

The variables were described using mean (x̅) and standard deviation (SD). Data normality was assessed using the Shapiro-Wilk test, and homogeneity of variances was assessed using Levene’s test. A two-way repeated measures analysis of variance (ANOVA) was used to evaluate the effects of time (pre- and post-intervention) and group (PDST, PIST, and CON) on TT, IRL, PT, CMJ, NF, GCT, and VO. When necessary, Bonferroni post hoc comparisons were performed to identify specific differences between time points and groups. Effect size was assessed using partial eta squared (η²p) for the ANOVA, and interpreted as small (≥ 0.01 and < 0.06), medium (≥ 0.06 and < 0.14), and large (≥ 0.14) [37]. Although not specified in the initial statistical plan, complementary analyses, such as 95% confidence intervals (CI) for the mean differences and Cohen’s d, were calculated as standardized measures of effect size for within-group comparisons. Effect size interpretation followed Cohen’s [37] guidelines: small (≥ 0.2 and < 0.5), moderate (≥ 0.5 and < 0.8), and large (≥ 0.8). Additionally, percent changes between time points were analyzed using analysis of covariance (ANCOVA), considering group as the factor and weekly running volume as a covariate only for TT, for which Pearson’s r was greater than 0.40. For the remaining variables, one-way ANOVA with the Bonferroni correction for multiple comparisons and Cohen’s d effect size between groups were used. Correlations between percent changes in PT and the percent changes in other variables were analyzed using partial correlations, controlling for weekly running volume as a covariate in the models. Pearson’s correlation coefficient (*r*) was used, and classified as very weak (0.00 ≤ |*r*| < 0.20), weak (0.20 ≤ |*r*| < 0.40), moderate (0.40 ≤ |*r*| < 0.60), strong (0.60 ≤ |*r*| < 0.80), and very strong (0.80 ≤ |*r*| ≤ 1). All analyses were conducted using Jamovi software (version 2.3.8, Sydney, Australia) and GraphPad Prism (version 10.2.2, California, USA), considering a significance level of *p* < 0.05. All randomized participants who completed post-intervention assessments were included in the analysis, except for the control group in the partial correlation analyses (n = 23), where only the intervention groups were considered.

## Results

The runners in the PDST, PIST, and CON groups exhibited similar characteristics in terms of age, body composition, weekly running mileage, and VO_2max_. Table 1 presents the sample characteristics.

**Table 1:** Sample characterization.

Variables	Mean ± SD			
	PDST (n = 11)	PIST (n = 12)	CON (n = 11)	*p*
Age (years)	18.9 ± 0.8	19.1 ± 0.7	19.2 ± 0.6	0.498
Body weight (kg)	68.9 ± 6.7	69.7 ± 9.2	67.9 ± 7.8	0.855
Height (cm)	176 ± 5	175.8 ± 5.6	173.1 ± 5.4	0.393
BMI (kg/m^2^)	22.2 ± 1.8	22.5 ± 2.7	22.7 ± 3	0.410
Weekly mileage (km)	16.8 ± 3.5	17 ± 3.2	17.3 ± 3.1	0.829
VO_2max_ (mL.kg^-1^.min^-1^)	52.1 ± 3.1	51 ± 2.4	51.5 ± 2	0.588

PDST = predominantly dynamic strength training, PIST = predominantly isometric strength training, CON = control, SD = standard deviation, BMI = body mass index, VO_2máx_ = maximal oxygen consumption,*p* = significance level.

The two-way repeated measures ANOVA revealed no significant differences between groups (*p* > 0.05) but indicated a significant main effect of time across all variables (*p* < 0.05), reflecting improvements after four weeks. The time × group interaction was significant only for PT (*p* = 0.007; *η²p* = 0.28) and CMJ (*p* = 0.001; *η²p* = 0.36), both indicating large effect sizes. Bonferroni post hoc analysis revealed significant differences only between the PDST and PIST groups (p < 0.05), suggesting improvements in isometric strength (PT) and neuromuscular capacity (CMJ). The CON group showed no significant changes. Table 2 details the ANOVA results and complementary within-group analyses (mean, SD, 95% CI, and Cohen’s *d*).

**Table 2:** Analysis of the effects of the intervention on performance, strength, fatigue, and running economy.

		TT	IRL	PT	CMJ	NF	GCT	VO
		(s)	(UA)	(Nm)	(cm)	(%)	(ms)	(cm)
**PDST** **(*n* = 11)**	**Pre**	808.0 ± 76.9	115.3 ± 16.2	222.0 ± 61.1	34.4 ± 4.6	1.1 ± 3.7	239.2 ± 8.8	10.6 ± 1.2
	**Post**	786.0 ± 73.5	108.7 ± 17.0	245.3 ± 49.4	36.2 ± 4.4	3.5 ± 4.5	233.2 ± 9.6	10.1 ± 1.5
	**95% CI**	−46.8; 2.8	−13.3; 0.0	10.8; 35.9	1.1; 2.4	−5.6; 0.7	−12; 0.0	−0.9; −0.1
	***d***	−0.2	−0.3	0.4	0.3	0.5	−0.6	−0.3
**PIST** **(*n* = 12)**	**Pre**	832.6 ± 64.9	120.3 ± 14.3	225.9 ± 52.1	34.1 ± 4.0	2.1 ± 2.5	244.5 ± 9.7	10.5 ± 1.2
	**Post**	810.8 ± 73.7	112.7 ± 15.9	255.6 ± 49.0	35.4 ± 3.6	3.0 ± 3.1	236.9 ± 8.9	9.8 ± 1.0
	**95% CI**	−41.4; −2.1	−13.8; −1.5	18.4; 40.9	0.7; 1.9	−3.1; 1.3	−11.9; −3.3	−1; −0.2
	***d***	−0.3	−0.5	0.5	0.3	0.3	−0.8	−0.6
**CON** **(*n* = 11)**	**Pre**	818.0 ± 47.1	119.0 ± 11.7	232.4 ± 45.0	33.8 ± 3.7	2.1 ± 3.1	241.3 ± 8.1	10.7 ± 0.9
	**Post**	810.3 ± 58.0	115.6 ± 11.7	237.3 ± 44.7	34.0 ± 3.7	2.7 ± 2.6	238.8 ± 10.7	10.4 ± 1.0
	**95% CI**	−17; 1.6	−10.8; 4.0	−6.5; 16.3	−0.3; 0.8	−2.5; 1.2	−5.4; 0.3	−0.6; 0.0
	***d***	−0.1	−0.2	0.1	0.0	0.2	−0.2	−0.3
**Group main effect**	**F (2, 31)**	0.43	0.44	0.07	0.33	0.03	0.86	0.31
	***p***	0.655	0.650	0.934	0.722	0.970	0.431	0.737
	***η*** ^***2***^ ***p***	0.03	0.03	0.00	0.02	0.00	0.05	0.02
**Time main effect**	**F (1, 31)**	11.86	11.33	39.94	51.21	4.21	20.66	25.41
	***p***	0.002	0.002	<0.001	<0.001	0.049	<0.001	<0.001
	***η*** ^***2***^ ***p***	0.28	0.27	0.56	0.62	0.12	0.40	0.45
**Time x group Interaction**	**F (2, 31)**	0.89	0.53	5.94	8.67	0.71	1.62	0.87
	***p***	0.421	0.592	0.007	0.001	0.502	0.215	0.429
	***η*** ^***2***^ ***p***	0.05	0.03	0.28	0.36	0.04	0.09	0.05

Notes: Values reported represent mean ± standard deviation. The alpha level was set at *p* < 0.05 for statistical significance.

PDST = predominantly dynamic strength training, PIST = predominantly isometric strength training, CON = control, TT = time trial, IRL = internal running load, PT = peak torque, CMJ = countermovement jump, NF = neuromuscular fatigue, GCT = ground contact time, VO = vertical oscillation, CI = confidence interval, *d* = Cohen’s effect size (small ≥ 0.2 and < 0.5, moderate ≥ 0.5 and < 0.8, and large ≥ 0.8), F = ANOVA value (degrees of freedom), *p* = significance level of two-way repeated measures ANOVA, *η^2^p* = partial eta squared (small ≥ 0.01 and < 0.06, medium ≥ 0.06 and < 0.14, and large ≥ 0.14).

**Fig 4:**
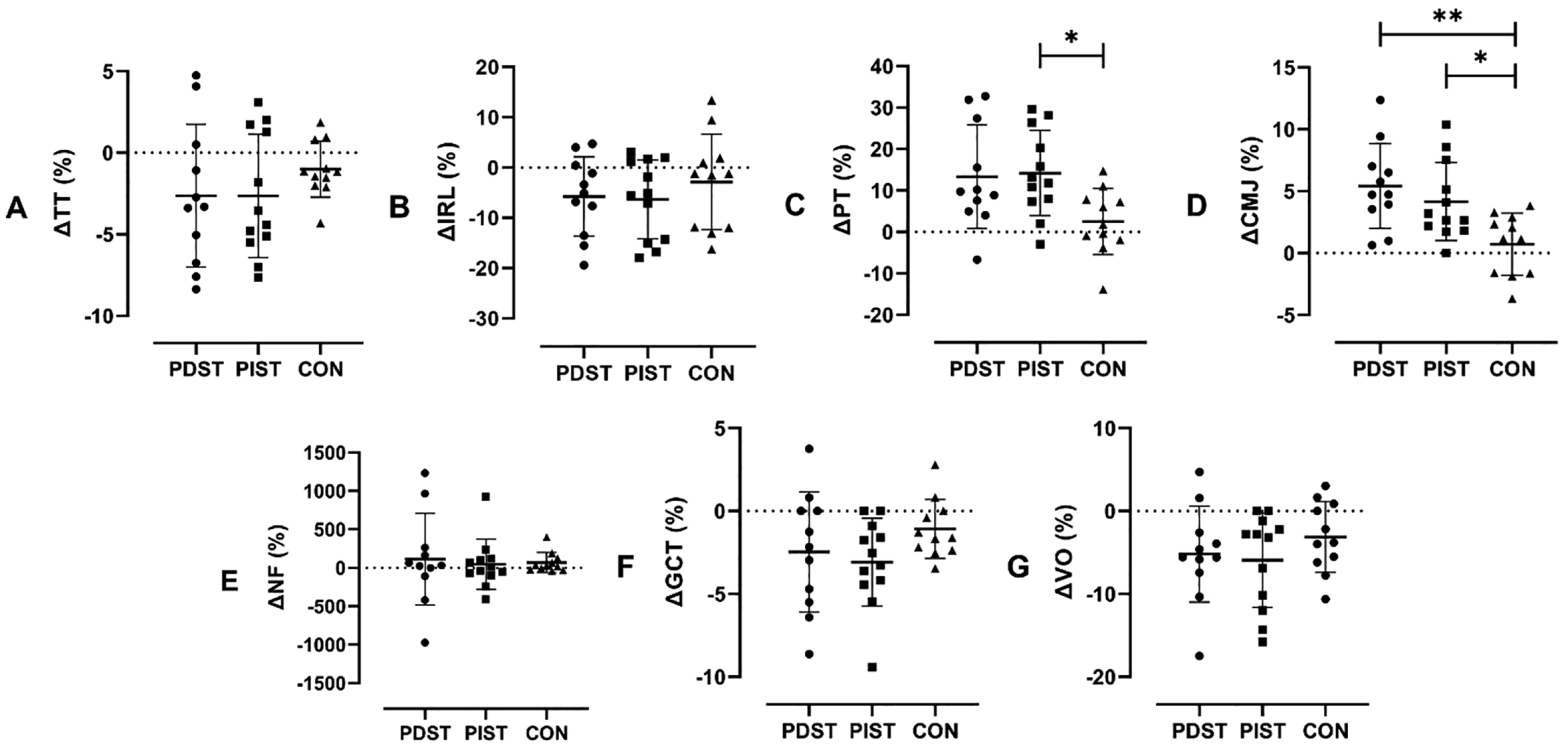
Pre-post percentage changes (mean ± SD) among the study variables. **A) TT, B) IRL, C) PT, D) CMJ, E) NF, F) GCT, and G) VO.** Indicates a statistically significant difference compared to the CON group *(*p* < 0.05), **(*p* < 0.01). CON =control, PDST = predominantly dynamic strength training, PIST = predominantly isometric strength training, TUT = time under tension, TT = time trial, IRL = internal rating load, PT = peak torque, CMJ = countermovement jump, NF = neuromuscular fatigue, GCT = ground contact time, VO = vertical oscillation.

To deepen the comparative analysis between groups—especially regarding percent means, individual participant distribution, and magnitude of changes—percent change (Δ%) was used as a complementary measure. For ΔPT, the group means were PDST: 13.3 ± 12.4%, PIST: 14.2 ± 10.3%, CON: 2.5 ± 7.9%. Bonferroni post hoc analysis revealed large effect sizes between PDST and CON, as well as between PIST and CON (*p* = 0.063; *d* = 1.04 and *p* = 0.034; *d* = 1.12, respectively). For ΔCMJ, the means were PDST: 5.4 ± 3.4%, PIST: 4.1 ± 3.1%, CON: 0.7 ± 2.5%, with large effect sizes (*p* = 0.003; *d* = 1.54 and *p* = 0.034; *d* = 1.13, respectively). Moderate but non-significant effects were found for ΔTT (PDST: −2.8 ± 4.3%, PIST: −2.6 ± 3.8%, CON: −0.8 ± 1.8%) between PDST and CON, and PIST and CON (*p* = 0.291; *d* = 0.73 and *p* = 0.355; *d* = 0.67, respectively), as well as for ΔGCT (PDST: −2.4 ± 3.6%, PIST: −3.09 ± 2.6%, CON: −1.08 ± 1.7%) (*p* = 0.759; *d* = 0.50 and *p* = 0.279; *d* = 0.72, respectively). However, for ΔTT, ANCOVA indicated that weekly running mileage had a significant effect on the model [F(1,30) = 21.19; *p* < 0.001; *η²* = 0.39], suggesting that this variable may have influenced the results. For ΔVO, the group means were PDST: −5.1 ± 5.7%, PIST: −5.9 ± 5.7%, CON: −3.1 ± 4.2%, with a moderate effect observed only between PIST and CON (*p* = 0.648; *d* = 0.53). For ΔITR and ΔNF, the group means were respectively: ΔIRL – PDST: −5.7 ± 7.8%, PIST: −6.2 ± 7.6%, CON: −2.8 ± 9.4%; ΔNF – PDST: −112.4 ± 595.8%, PIST: −45.4 ± 324.4%, CON: −67.6 ± 129.9%, with only small effects (*d* < 0.5) observed between groups. No moderate or large effects were observed between PDST and PIST in any variable. Graphical results of the ANCOVA (ΔTT) and one-way ANOVA (ΔIRL, ΔPT, ΔCMJ, ΔNF, ΔGCT, and ΔVO) are presented in Fig 4A–G.

**Fig 5:**
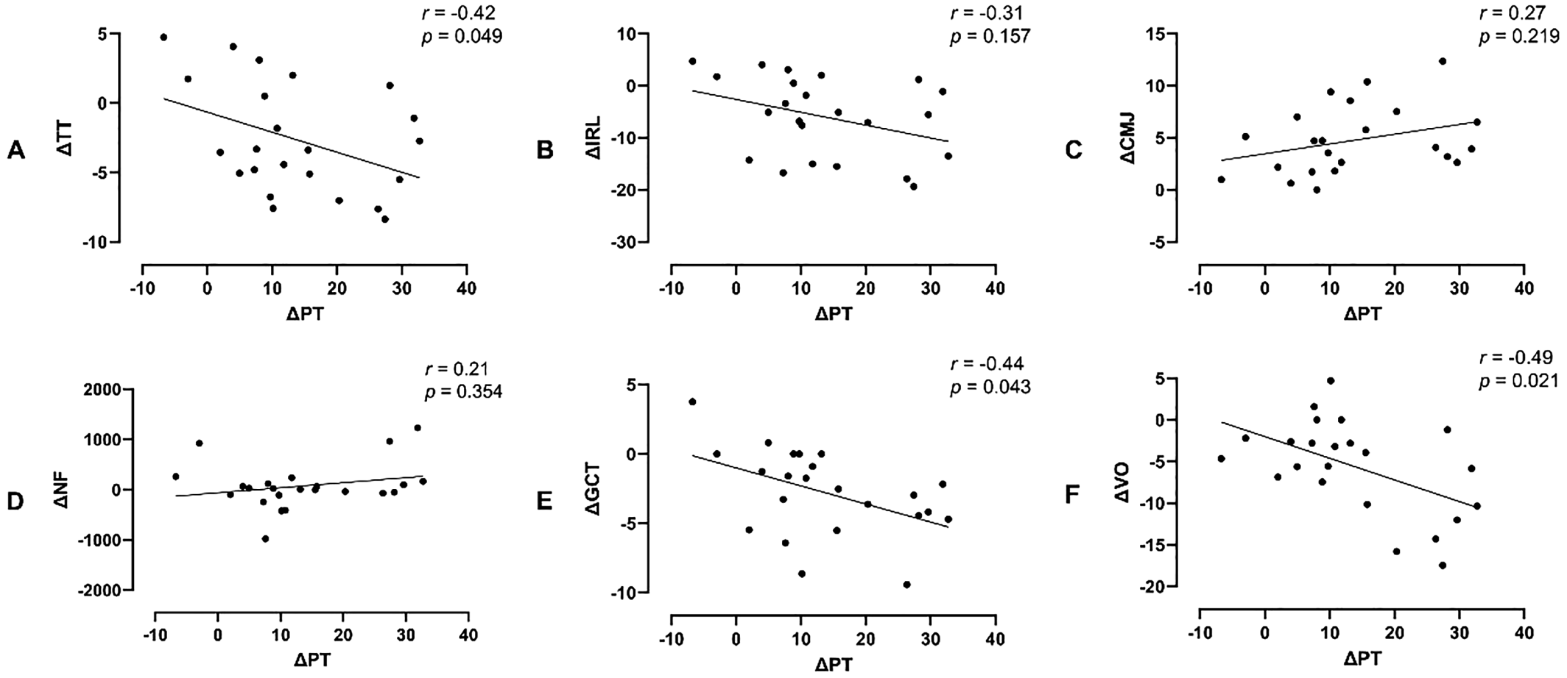
Partial correlations between PT variation and other variables in PDST and PIST (n = 23). **A)** PT and TT, **B)** PT and IRL, **C)** PT and CMJ, **D)** PT and NF, **E)** PT and GCT, **F)** PT and VO. PDST = predominantly dynamic strength training, PIST = predominantly isometric strength training, PT = peak torque, TT = time trial, IRL = internal rating load, CMJ = countermovement jump, NF = neuromuscular fatigue, GCT = ground contact time, VO = vertical oscillation, *r* = Pearson’s correlation coefficient, *p* =significance level of partial correlation.

Pearson correlations controlled for weekly running mileage in the PDST and PIST groups (n = 23) indicated moderate inverse correlations only between ΔPT and ΔTT [*r* = −0.42 (95% CI = −0.78; −0.05); *p* = 0.049], ΔPT and ΔGCT [*r* = −0.44 (95% CI = −0.79; −0.08); *p* = 0.043], and ΔPT and ΔVO [*r* = −0.49 (95% CI = −0.82; −0.15); *p* = 0.021]. All correlations between PT percent changes and the other variables are shown in Fig 5A–F.

## Discussion

The present study aimed to compare the effects of ST with high-TUT on performance-related variables in runners, particularly in predominantly dynamic and isometric actions. The main findings showed that PIST resulted in an improvement in ΔPT compared to CON. Additionally, PDST and PIST improved ΔCMJ post-intervention, with no differences between them. Furthermore, considering the similarity of the high-TUT protocols across the findings, moderate inverse correlations were observed for ΔPT and ΔTT, ΔPT and ΔGCT, and, finally, between ΔPT and ΔVO.

These findings partially confirm our initial hypothesis. Indeed, neuromuscular improvements were observed at the end of the intervention. These findings partially confirm our initial hypothesis. Indeed, neuromuscular improvements were observed at the end of the intervention, even though no significant differences in ΔPT were detected between PDST and CON, although large effects were observed in the multiple comparisons.

These results are consistent with the recommendations of Mang et al. [1], based on previous evidence [21,38–40], which suggests that ST with low intensities and slow execution tempo may induce positive neuromuscular adaptations, especially when the TUT per repetition is around 7 seconds, thereby increasing the total TUT per set.

Notably, PIST showed statistical significance compared to CON for ΔPT. Indeed, exercises such as squats and unilateral lunges were performed between 100° and 110° of knee flexion in PIST, while PT was assessed at 70°. Despite the angular difference of 30° to 40°, the more favorable results for the isometric group can be explained by the greater compatibility between the type of contraction trained and the evaluation. Although the literature indicates that isometric strength transfer occurs up to ±20° from the trained angle [41,42], it is also likely that sustained execution at nearby joint angles favored specific adaptations to PIST, as pointed out by Folland et al. [43], contributing to its slight superiority over PDST. In addition, the high-TUT and continuous quadriceps activation during the exercises may have potentiated these adaptations.

Regarding ΔCMJ, increases in jump capacity with large effect sizes were observed for PDST and PIST vs. CON. These results are also aligned with studies that have demonstrated improvements in jump performance after ST, emphasizing both dynamic and isometric actions [14,44,45]. Even without differences between the protocols, the more pronounced effect for PDST (*d* = 1.54) is consistent with the literature, primarily because improvements in jump performance have been attributed to greater eccentric control promoted by prolonged TUT (40). Notably, ballistic actions show greater performance [14]. Thus, although PDST did not emphasize the rapid reactive actions of the SSC, the higher TUT during the eccentric phase may have facilitated greater muscle activation and the potential availability of cross-bridges at the onset of the concentric phase. This mechanism represents an alternative pathway to the traditional elastic potentiation of the SSC, highlighting the role of eccentric control in enhancing jump performance [44,46]. Additionally, neuromuscular adaptations related to intra- and intermuscular coordination in the runners of the present study may also have contributed to the improvement in jump performance in both protocols.

In this sense, the improvements in strength and vertical jump can also be explained by neural mechanisms associated with training under high-TUT. The prolonged muscle activation time during each repetition, combined with execution to near failure, promotes a physiological environment characterized by the accumulation of metabolites (lactate, H ⁺ , inorganic phosphate), which favors the additional recruitment of higher-threshold motor units, including type II fibers [47,48]. According to Henneman’s size principle [49], such recruitment would only occur at high intensities; however, under metabolic fatigue, this stimulus can be achieved. This recruitment pattern has been identified as one of the primary mechanisms through which high-TUT ST, even with reduced loads, can generate relevant neural adaptations [38,50].

On the other hand, it was expected that high-TUT protocols would result in significant improvements in TT compared to CON, given that this variable was defined as the primary outcome in the present study. In this context, although our results do not align with previous studies reporting improvements in the running performance of runners subjected to ST [2,14,51], it is essential to highlight that these investigations did not adopt high-TUT protocols, which may explain the discrepancies observed. Moreover, these studies were conducted with interventions lasting between 6 and 14 weeks, whereas the present study lasted only 4 weeks. This choice aimed to minimize the influence of peripheral aerobic adaptations, such as angiogenesis and mitochondrial biogenesis, which could attenuate or mask the neuromuscular effects of strength protocols [1].

Nevertheless, despite the short duration, the moderate inverse correlation between ΔPT and ΔTT suggests that strength gains may contribute, to some extent, to improved running performance. A similar result was observed by Lum et al. [14], who also identified a moderate correlation between strength and performance in runners subjected to dynamic and isometric training, though without a focus on elevated TUT. Conversely, when comparing the *η²p* of the time × group interaction between the study of Lum et al. [14] (*η²p* = 0.47) and the present study (*η²p* = 0.05), a relevant discrepancy is observed. In addition to methodological differences between the protocols and the race distance (2400 m vs. 3000 m), it is possible that the greater homogeneity of the sample in this study (composed solely of men, with little ST experience and controlled weekly mileage) limited variability, allowing a more targeted analysis of the effects of the protocols on running performance.

However, although some of the studies mentioned above have reported improvements in running performance following strength-training interventions, other investigations have shown a different pattern. Specifically, despite observing short-term neuromuscular enhancements, these studies did not identify changes in running mechanics or performance outcomes after eight weeks of training, even when using interventions different from those applied in the present study [52,53]. These findings suggest that neuromuscular adaptations, although relevant, are not sufficient, by themselves, to produce detectable changes in running performance over such short periods. Therefore, the absence of significant improvements in performance observed in this study likely reflects the fact that endurance performance depends on a broad set of determinants, such as threshold-related velocities, velocity at maximal oxygen uptake (vVO_₂max_), running economy, and pacing ability, that may not be sufficiently modified in short-term interventions, even when neuromuscular adaptations are present [54].

Additionally, IRL was used to investigate the relationship between TT performance and RPE, aiming to quantify perceptual changes associated with tolerance and adaptation to intense effort. It was expected that high-TUT ST would reduce IRL, reflecting a lower perceptual cost related to sustaining performance. However, no statistically significant reductions were observed among the groups throughout the interventions. A possible explanation is the high intensity of the test, which tends to stabilize RPE at high levels regardless of peripheral physiological adaptations. In fact, recent literature suggests that RPE is predominantly regulated by central mechanisms, with limited influence from peripheral changes such as muscular adaptations [29,55,56]. This explains why IRL did not decrease even with neuromuscular improvements. Moreover, the absence of correlation between ΔPT and ΔIRL reinforces this interpretation, indicating that even with increased muscle strength, the effort perception associated with performance remained unchanged.

In the biomechanical analysis of GCT and VO, although no statistically significant differences were observed, the medium effect sizes between PIST and CON indicate directional changes that were not sufficient to support meaningful between group differences. Reductions in such measures may primarily stem from musculotendinous adaptations, such as achieving an adequate level of tendon stiffness, since tendons with appropriate stiffness allow for faster and more efficient force transmission (51,52), thereby favoring shorter ground contact times [15]. It has also been shown that increases in muscle strength after elevations in tendon stiffness are accompanied by high-TUT ST programs [57], particularly in isometric exercises [58,59].

These findings suggest that predominantly isometric training performed under high-TUT may influence variables associated with running economy. However, because stiffness was not directly measured in the present study, such interpretations remain speculative. Moreover, unlike previous studies that have reported significant improvements in stiffness or running economy following isometric training, we did not observe meaningful changes in GCT or VO. Among the factors that may explain these discrepancies, differences in intervention duration appear to strongly influence the effects observed in this study. It is also possible that adaptations occurred primarily at the neuromuscular level (e.g., motor unit recruitment or intermuscular coordination), but without sufficient magnitude to produce statistically detectable changes in the mechanical variables assessed. Taken together, these aspects may explain why our results differ from previous findings.

On the other hand, considering the effects of the interventions on NF, it was observed that high-TUT protocols did not result in marked changes in this parameter, as indicated by the small impact observed between groups. This suggests that the different actions did not induce significantly different levels of fatigue compared to the CON group. Still, both high-TUT protocols induced NF, especially PDST. This can be explained by the fact that predominantly eccentric actions impose greater mechanical stress on muscle fibers during lengthening under tension, resulting in greater muscle damage and a more extended recovery period compared to concentric and isometric contractions [60–63]. However, as observed in Fig 4E, although the groups did not show significant differences, this supports our initial hypothesis; however, it cannot be stated that high-TUT modulates NF, due to high interindividual variability, particularly in PDST. Moreover, the CON group presented a considerable level of fatigue, possibly due to not having been subjected to the intervention, making it challenging to isolate protocol-specific effects.

In this sense, the magnitude of fatigue in the groups could be conditioned by improvements in TT performance after the training sessions, given the high demand of this trial and the subsequent jump assessment. This can be inferred because, by improving neuromuscular aspects, one would expect greater efficiency in force production mechanisms (such as type II fiber recruitment), resulting in greater responsiveness to high-intensity demands [6,64]. However, despite this expectation, no significant correlation was observed between ΔNF and ΔTT [r = −0.22 (95% CI = −0.58; 0.22); *p* = 0.336] regarding the interventions, indicating that NF was not highly associated with running performance. Thus, the observed effects appear more closely related to the specifics of the high-TUT protocols in inducing fatigue, albeit to a lesser extent.

Additionally, evidence comparing contractions performed at fast versus slow velocities suggests that protocols with slow velocities (and consequently high-TUT per set), such as those used in this study, tend to induce a lower temporal course of acute NF and residual muscle damage than those focusing on explosive actions [9,10]. This suggests that high-TUT protocols may be beneficial for managing fatigue and muscle damage in runners, serving as an introductory strength training strategy. Thus, such protocols can be applied by coaches during short adaptation periods, preparing runners for subsequent phases of greater intensity, such as plyometrics or progressive use of external loads.

Clearly, in this study, comparison with previous studies using high-TUT in runners was challenging, as there is a scarcity of research with similar protocols in the current scientific literature, mainly since the focus of interventions in runners has concentrated on aerobic, plyometric, or conventional strength methods [6,65]. However, the present study aimed to provide an overview of high-TUT on variables related to performance and neuromuscular function, offering initial insights into the effects of this training strategy in runners.

Moreover, although running performance did not improve over the four weeks, longer interventions would likely yield positive results for high-TUT protocols, provided that peripheral aerobic adaptations are controlled and distinguished from neuromuscular adaptations. Still, our findings demonstrated that high-TUT ST, combined with endurance training, promotes gains in maximal strength and jump capacity. Such effects had already been observed in previous studies with methodologies distinct from high-TUT [14]. This reinforces the applicability of high TUT as an effective strategy, although further evidence is needed to confirm its effectiveness. These results also reinforce the training specificity principle [14], evidenced in this study by the more prominent improvement in PT in PIST and in CMJ in PDST. From a practical standpoint, high-TUT training may be considered a complementary approach to optimize neuromuscular qualities relevant to running performance. However, such potential benefits may become more evident with longer-duration interventions or with larger sample sizes, which are capable of detecting smaller yet significant changes in running outcomes.

The significant improvements observed in peak torque for PIST and in countermovement jump for both PDST and PIST, although not immediately translating into measurable running performance gains over the four-week intervention, hold clear practical relevance. Increases in maximal strength and jump capacity are strongly associated with enhanced neuromuscular function, which underpins more efficient force production, better eccentric control, and improved potential for energy transfer during running [6].

From a physiological perspective, these neuromuscular adaptations may also influence the overall energetics of distance running by improving mechanical efficiency. Efficiency during locomotion emerges from the interaction between the metabolic cost of movement and the mechanical work effectively transmitted through the musculoskeletal system, being modulated by mechanisms such as elastic energy reutilization [66]. In this sense, the gains observed in strength and jump performance may reflect improvements in the ability to store and release elastic energy and in transmission efficiency, potentially reducing the relative mechanical work required at a given running intensity and, over time, lowering metabolic cost. Although these adaptations were not reflected in improved 3000 m TT performance (likely due to the short duration of the intervention), they represent foundational adaptations that, in the long term, could contribute to better running economy, reduced injury risk, and enhanced performance. Thus, even modest neuromuscular improvements, as observed here, can be considered clinically meaningful for practitioners aiming to implement high-TUT strength protocols as part of a comprehensive and energy-efficient running training program.

### Limitations

Some limitations should be considered when interpreting the results of this study. First, the sample size calculation was based solely on the primary outcome, disregarding the specific variability of secondary variables. Although adequate for the main objective, this may have reduced the sensitivity to detect differences in other outcomes. Future studies should consider sample size calculations specific to each primary variable to maximize statistical power across different outcomes [67]. Additionally, although we initially planned to recruit 36 participants, the final sample consisted of 34 individuals due to losses that occurred during the study. This reduction was small and kept the sample size close to the planned number, minimizing its impact on the pre-established statistical power. Nevertheless, we acknowledge that this decrease represents a limitation, as larger sample sizes could increase the ability to detect subtle effects.

Second, the sample included only male runners because the military unit from which participants were recruited was predominantly composed of men available during the data collection period. Additionally, restricting the sample to one sex helped reduce variability associated with sex-specific physiological factors, such as hormonal fluctuations, strength levels, fatigue responses, and running economy, which could introduce additional noise into a short-term intervention. However, we acknowledge that this choice limits generalizability, and future studies should investigate whether high-TUT protocols produce similar neuromuscular and performance responses in female runners, given the known physiological and biomechanical differences between sexes.

Third, muscle strength was assessed exclusively through the isometric peak torque of the knee extensors. Although methodologically justifiable, given the high demand on the quadriceps in the protocols, this approach, restricted to a single muscle group, limits the overall understanding of neuromuscular adaptations. Thus, although the observed gains may indicate improvements in motor patterns and synergistic muscles (such as the gluteals, hamstrings, and plantar flexors), this inference could not be directly confirmed. It is recommended that future studies incorporate dynamic, isokinetic, and multisegmental assessments for a broader characterization of the effects of high-TUT on the neuromuscular system [13].

Fourth, we used GCT and VO as biomechanical variables to estimate the degree of musculotendinous stiffness adaptation. Even though studies show a relationship between these variables and stiffness [15,58], the indirect assessment limits the inferences. Future studies may include instruments that guarantee greater precision, such as ultrasonography with elastography or dynamometry.

Fifth, CMJ was used as a measure of NF induced by high-TUT protocols; however, future studies may include biochemical markers, such as creatine kinase (CK), to complement the analysis and provide a more comprehensive assessment, as this may indicate whether fatigue is associated with structural muscle damage.

Finally, the duration of the intervention, limited to four weeks, represents an important limitation of the present study. This relatively short period may not have been sufficient for broader adaptations, especially those related to running performance and running economy, to manifest in a measurable way. Although we expected that improvements in neuromuscular parameters might translate into performance gains, this was not observed. Therefore, we suggest that future investigations examine high–TUT protocols over longer intervention periods to determine whether such neuromuscular adaptations can, in fact, lead to meaningful improvements in running performance.

## Conclusion

This study demonstrated that both PDST and PIST promoted similar improvements in PT and CMJ compared to the CON group, albeit with specific responses to each intervention. These findings reinforce the influence of high-TUT as a determining factor in promoting neuromuscular adaptations, regardless of the predominant type of contraction. However, caution is needed when interpreting these results and avoiding generalizations, considering the sample size and the specific profile of the participants. Future studies should investigate the effects of high-TUT in runners on performance variables over more extended intervention periods (e.g., > 6 weeks), particularly while controlling for peripheral aerobic adaptations.

In addition, the results show that high-TUT is a promising strategy even in individuals with less familiarity with ST. In more experienced runners, specific adjustments in intensity and volume may be necessary. Moreover, due to its low fatigue generation, the application of high-TUT protocols in runners appears beneficial during phases of higher running volumes or as preparation for methods that demand greater mechanical stress and specificity, such as plyometric training, although further studies are still needed.

## Supporting information

S1 File Checklist. (PDF)

S2 File Study protocol. (PDF)

S3 File Dataset. (XLSX)
