# Supplementary material for: Effect of high time under tension strength training on different muscular actions in the performance of runners: A randomized controlled trial
Source: PLoS One. 2026 Feb 9;21(2):e0342428. doi: 10.1371/journal.pone.0342428 (PMC12885319; doi:10.1371/journal.pone.0342428)
Supplement: S2 File — (PDF) [file pone.0342428.s002.pdf]

STUDY PROTOCOL

1. SUMMARY

Recreational running (RR) is one of the most popular sports among both performance and recreational athletes, making it one of the most accessible modalities due to its low cost and numerous positive health and physical conditioning effects. In relation to RR practice, strength training (ST) has been employed to enhance muscle strength gains, reduce ground contact time, stride length, and step frequency, and improve running performance by increasing muscle capillarization, mitochondrial density, and maximum oxygen consumption (VO2max). However, much of the ST prescription has been based on high volumes and low intensities. Current scientific evidence suggests that low repetitions and control of movement cadence through concentric, eccentric, and isometric muscle actions in ST can produce the same effects as traditional ST for running, a factor associated with time under tension (TUT). Regarding recreational road runners, the impact of TUT on running performance has been little investigated. Therefore, this study aims to evaluate the chronic effect of strength training with time under tension on the performance of recreational runners. We believe that isometric ST influences running performance and produces greater adaptive responses in physiological and biomechanical aspects when compared to concentric/eccentric ST.

2. INTRODUCTION

Running has become popular worldwide (CHANG; SHIN; CHEN, 2012). In the recreational context, running offers benefits associated with lifestyle changes and improved health, being considered a sport that provides excellent cost-effectiveness (LEE et al., 2017). However, as running becomes a habit, it is common for runners to seek rewards through practice, such as participating in competitions and events related to the sport (GAUFFIN et al., 2019; TEIXEIRA, 2012). Consequently, this results in a constant pursuit of running performance and physical achievement across various distances.

In long-distance races (≥3000 meters), maximum oxygen consumption (VO2max), lactate threshold (LT), and running economy (RE) are physiological and biomechanical factors that determine running performance (ANDERSON, 1996; MIDGLEY, MCNAUGHTON, JONES, 2007). Additionally, this type of running is also associated with central physiological adaptations (greater stroke volume, cardiac output, and myocardial efficiency) and peripheral adaptations (higher capillary density and mitochondrial density) that respectively improve cardiovascular capacity and the extraction of oxygen from the blood to the skeletal muscle (HAWLEY et al., 2014; MACINNIS, GIBALA, 2017; MANG et al., 2022). These factors are crucial for

maintaining aerobic capacity and sustaining prolonged efforts.

Despite the predominance of aerobic characteristics in this type of running, studies affirm that anaerobic and neuromuscular components also influence performance (NOAKES, MYBURGH, SCHALL, 1990; PAAVOLAINEN et al., 1999), as running requires repeated force production with each step (MIKKOLA et al., 2011). In this sense, strength training (ST) has contributed to running performance by promoting better muscle-tendon stiffness, stretch-shortening cycle, intra- and intermuscular coordination, and force levels—factors that result in reduced neuromuscular fatigue, improved force application in longer runs, and consequently, greater running economy (BLAGROVE et al., 2020; GÄBLER et al., 2018; PRIETO-GONZÁLEZ, SEDLACEK, 2022; RØNNESTAD, MUJIKA, 2014).

It is also known that different types of strength training (maximal strength training, explosive strength training, and endurance training) associated with different volumes and intensities promote improvements in EC, subjective perception of effort (SPE), and strength in recreational runners (DOMA; DEAKIN, 2014; FERRAUTI, BERGERMANN, FERNANDEZ-FERNADEZ, 2010; TAIPALE et al., 2010). In this regard, Mang et al. (2022) report that a variable of strength training that has stood out in the literature regarding its influence on aerobic performance and force production is time under tension (TUT).

TST is known for controlling repetition duration through concentric, eccentric, and isometric muscle actions (CINTINEO et al., 2018; MOOSES et al., 2021). Studies have confirmed that training protocols with greater emphasis on the concentric and eccentric phases (e.g., 3 seconds of concentric phase, 3 seconds of eccentric phase, and 1 second of recovery) promote significant gains in muscle strength at lower intensities (30-50% of 1RM), stimulating neuromuscular adaptations (force production and muscular endurance) and metabolic stress (greater aerobic fitness) in the long term, being considered the primary type of TST (DAVIES et al., 2017; MANG et al., 2022; TANIMOTO, ISHII, 2006). Additionally, in running, eccentric and concentric forces have been associated with greater actions during the initial contact phase with the ground (support phase) and the push-off phase (ground reaction force), respectively (LI et al., 2019; SUNDBY, GORELICK, 2014).

On the other hand, other studies suggest that training protocols emphasizing isometry (e.g., 1 second of concentric phase, 5 seconds of isometric phase, and 1 second of eccentric phase) can also produce the same effects as concentric/eccentric training (GENTIL, OLIVEIRA, BOTTARO, 2006; MANG et al., 2022). Furthermore, they may lead to better performance and associated increases in maximum voluntary isometric contraction strength (MVIC), Achilles tendon stiffness (ATS), higher flight rate in counter-movement jumps (CMJ), and reduced ground contact time (GCT) dynamically, that is, during a long-distance time trial (e.g., 3000m) (ARAMPATZIS et al., 2007; BOGDANIS et al., 2019; FLETCHER, ESAU, MACINTOSH,

2010; KUBO et al., 2012; LICHTWARK, BOUGOULIAS, WILSON, 2007).

Despite the importance of strength training (ST), in the context of recreational runners, performing two types of modalities simultaneously (running and ST) has not been common, as most have family and professional commitments that do not allow for such regularity, especially in ST sessions (FESTA et al., 2019). Furthermore, although it has been reported that both concentric/eccentric strength training and isometric strength training improve running performance at low intensities, so far, there are no studies that have compared both types of ST without the use of external load, that is, only with body weight. In this sense, the study is justified by the need to determine the best type of ST (concentric/eccentric or isometric) for runners, aiming for greater practicality and suitability to their reality, as well as increased applicability by coaches and running clubs regarding ST prescription.

Given this, the following question arises: What type of TST in the TF is capable of promoting better time trial running performance and also improving the associated physical performance, including muscular strength, reduction of muscle fatigue, perception of effort, and running dynamics? We believe that isometric strength training, compared to concentric/eccentric strength training, yields greater results in running performance.

3. OBJECTIVES

3.1. GENERAL

The objective of this study, therefore, is to compare the effect of strength training with different time under tension on the performance of recreational runners after a 4-week intervention.

3.2. SPECIFIC

- Compare the effect of 4 weeks of concentric/eccentric strength training versus isometric training on 3000 m time trial performance, PSE, and TCS.
- Compare the effect of 4 weeks of concentric/eccentric strength training versus isometric training on the peak torque of the knee extensor muscles.
- Compare the effect of 4 weeks of concentric/eccentric strength training versus isometric training on neuromuscular fatigue level measured by countermovement jump.

4. METHODOLOGY

SAMPLE

This is a proposal for a randomized clinical trial involving recreational male runners from

the city of Lagarto, Sergipe, Brazil. The runners will be recruited through a meeting to present the study's objectives. The inclusion criteria will include:

(1) age ≥ 18 years;

(2) run at least three times a week with an average weekly mileage of 12 km (≥3 days/week and ≥12 km/week);

(3) maintain an average running pace between 4:00 and 5:30 min/km over long distances (3000m);

(4) participate in a strength training program at least once a week (≥1 day/week) for at least three months;

(5) report absence of osteoarticular injury during the last three months that affected strength exercises and running;

(6) have at least one year of experience in long-distance races (3000 m-5000 m).

The exclusion criteria will include:

(1) perform physical exercises and/or consume alcohol 24 hours before the tests and intervention;

(2) consume caffeine 4 hours before the tests and the intervention.

Sample size calculation

The sample size calculation will be conducted a priori using G*Power software (version 3.1.9.7, University of Kiel, Kiel, Germany), adopting a significance level of $\alpha < 0.05$ and a statistical power ($1-\beta$) of 0.80. For this, an F-type statistical analysis (two-way repeated measures ANOVA) will be configured, considering the interaction between the factor group (IST vs. CEST vs. CON) and time (pre vs. post).

Research ethics

Participants will be informed in detail about the study's objectives and procedures, and they will be required to sign a form indicating their free and informed consent if they wish to participate in the research. The current study will be submitted to the Research Ethics Committee for Human Subjects at the Federal University of Sergipe (UFS), with data collection beginning after approval.

Experimental design of the study

The study will be conducted over a period of 6 weeks. Participants will be randomly assigned to three groups matched by 3000 m time trial performance (LI et al., 2019). An individual not involved in the research will be responsible for matching and randomization, using Euclidean

distance. Therefore, the groups will be divided as follows: isometric strength training (IST), concentric/eccentric strength training (CEST), and a control group (CON). For four weeks, runners in the IST and CEST groups will perform bodyweight strength training and TST, with a greater emphasis on the isometric and concentric/eccentric phases, respectively. Both groups will undergo two training sessions per week with 48-hour rest intervals between sessions, totaling eight sessions over the 4-week intervention. The weekly training schedule for both groups will be as follows: IST – every Monday and Wednesday; CEST – every Tuesday and Thursday; CON – no intervention. All participants will be advised to maintain their usual running routines during the week, except on strength training days. During all sessions, which will be held at the Tiro de Guerra 06-015, a Physical Education professional specialized in TF will be present to ensure the correct handling of the TST variable in each protocol.

Before (week 1) and after the intervention (week 6), the runners will visit the unofficial athletics track at TG 06-015. The first visit (pre-test) will be divided into two moments (pre- and post-3000 m time trial), consisting, respectively, of the pre-period: collection of personal data (medical history), anthropometric assessments (body mass, height, and Body Mass Index), and assessment of peak torque (PT) (BENTO et al., 2010). In the post-period, it will include: subjective perception of effort (RPE) (FOSTER, 2001), assessment of countermovement jump height (CMJ) (KNIHS, 2022), ground contact time (GCT) (LORIMER, WADER, PEARSON, 2016; ADAMS et al., 2016), and familiarization with strength training protocols, as suggested by Damasceno et al. (2015). After the 4-week intervention, the participants will return to TG 06-015 and perform an identical post-test to the pre-test.

Additionally, to avoid variations caused by the circadian cycle, pre-intervention, intervention, and post-intervention assessments will be conducted at the same shift and time, previously scheduled with the participants. Furthermore, participants will be asked not to engage in other strength training programs beyond those necessary for the current investigation (SEDANO et al., 2013), but to continue their regular running workouts.

Training protocols

Strength training protocols will focus on the lower limbs and will consist of 4 exercises (squats, unilateral lunges, hip elevation on the ground, plantar flexion), with four sets of 12 repetitions at an intensity equivalent to body weight (without external load) for each exercise (adapted from JOHNSON et al., 1997; LUCKIN-BALDWIN et al., 2021) and with the appropriate TST for each group, organized as follows: IST – cadence 1510 (2 seconds eccentric phase, 5 seconds isometric phase, and 2 seconds concentric phase) (GENTIL, OLIVEIRA, and BOTTARO, 2006; MANG et al., 2022); CEST – cadence 3130 (3 seconds concentric phase, 3 seconds eccentric phase, and 1 second isometry) (DAVIES et al., 2017; TANIMOTO, ISHII,

2006). At the end of each set, a 90-second rest interval will be observed. After each exercise, a 120-second recovery time will also be timed. Additionally, a metronome (Korg, model MA-2, Tokyo, Japan) (TOZEI et al., 2021) will be used to control the TST variable. Table 1 shows the structure of the sessions for each group.

Table 1. Characteristics of isometric strength training (IST) and concentric/eccentric strength training (CEST).

Protocol	Weekly sessions	Sets	Repetitions	Number of exercises	Cadence (E-I-C-I)
**IST**	2	4	12	4	1510
**CEST**	2	4	12	4	3130

Note. C: concentric; E: eccentric; I: isometric; s: seconds.

Body composition assessment

On the first visit, the mass and height of the volunteers will be measured as part of the sample characterization. Body mass and height will be measured using an analog scale with a stadiometer, with a precision of 0.1 kg (Filizola, MIC 2B/A Mechanical Anthropometry, 300 kilograms, Brazil).

3000m time trial

The 3000-meter time trial performance test will be conducted on the official TG 06-015 athletics track in two sessions (pre-intervention and post-intervention). The runners will perform the test individually. During the run, participants will be encouraged through clapping and motivational words. The participants' running times will be monitored in both trials using a digital stopwatch (Vollo Sports, model VL515, São Paulo, Brazil) and a GPS watch (Garmin Ltd, model Forerunner 245, Kansas, USA) (MOREIRA et al., 2013) to obtain more accurate data. The warm-up for the test will consist of 5 dynamic exercises: Frankenstein, Anfersen, small jumps, low skipping, and high skipping (adapted from ZOURDOS et al., 2012; YAMAGUCHI, TAKIZAWA, SHIBATA, 2015). At the end of the time trial, participants will not be informed about the number of laps on the track or their final time. Running performance will be recorded as the average running pace (minutes per kilometer).

Peak torque

The peak torque (PT) will be collected as the maximum isometric torque produced by the knee extensor muscles (quadriceps). The PT will be determined by multiplying the peak isometric force of the quadriceps (dominant leg) by the segment length, given by the distance between the

load cell attachment point and the central axis of the knee joint. The isometric force will be measured using a load cell (Kratos, model CZC500, São Paulo, Brazil) attached to an inextensible cord and positioned near the malleolus with Velcro. The runners will have three attempts to familiarize themselves with the instrument. Subsequently, they will perform three more maximum attempts, and the highest torque peak will be used for analysis. Participants will produce torque quickly and forcefully for a short period (around 2-3 seconds). At the end of each effort, a 1-minute rest interval will be observed (BENTO et al., 2010).

Counter Movement Jump (assessment of neuromuscular fatigue)

The assessment of the vertical jump with counter-movement will be conducted during the first visit (week 1) and the second visit (week 6) during the post-counterclockwise moments. The neuromuscular fatigue assessment will be performed using the flight time parameter (height of the jump in the air) (KNIHS, 2022). Regarding the jump procedure, the participant will start in an upright position, with feet placed shoulder-width apart on a mat and hands on the hips, performing a downward movement by flexing the knees to approximately 90° to jump as quickly as possible with maximum effort (BLAGROVE et al., 2019). The test will be conducted using a jump platform (Chronojump-Bosco System, Barcelona, Spain) connected to a computer software. For data analysis, the best result from three attempts, with a 1-minute interval between each attempt, will be considered.

Rating Perception of Effort

The Borg CR-10 Rating of Perceived Exertion scale (1982), adapted by Foster et al. (2001), will be used to assess RPE at two points during the time trial: after the first time trial and after the second time trial. Based on this, the internal load control of the race (CIC), proposed by Foster et al. (2001) and adapted from Sant'ana, Bara-Filho, and Vianna (2021), will be calculated and expressed in arbitrary units (AU). The following formula will be used:

CIC = total time of the time trial X RPE of the time trial

Ground contact time

The contact time with the ground variable (GCT) will be collected using a Garmin HRM-Run accelerometer (Garmin Ltd, Kansas, USA) along with a Garmin Forerunner 245 watch (Garmin Ltd, Kansas, USA). Both devices have validity and reliability in detecting running dynamics, especially GCT (ADAMS et al., 2016). Participants will be previously instructed and familiarized with the operation of the “start” and “stop” functions on the watch. The GCT will be expressed in milliseconds (ms), considering the average value during the time trial races 1 and 2.

5. POSSIBLE RISKS

Since this study involves human beings, potential issues (such as embarrassment, injuries, and other adverse events) may occur. Regarding the assessment of body composition, participants may experience slight discomfort due to the contact of the skinfold caliper with the evaluated person's skin. To minimize discomfort, the quality of the collection materials is considered, as well as the presence of a qualified Physical Education professional. Additionally, participants may feel some embarrassment after receiving the results of the anthropometric tests, which will be minimized by maintaining the confidentiality of their data. Participants are also exposed to risks associated with aerobic capacity assessments (a 3 km time trial run) and the experimental session (strength training), which may cause shortness of breath and muscle soreness as effects of physical effort and muscle force production, respectively, from both types of training. These can be minimized with the supervision of a Physical Education professional throughout the process, and additionally, by presenting a MEDICAL CERTIFICATE authorizing physical activity and granting permission to participate in the study.

6. PROVISIONS AND PRECAUTIONS

If the participant reports pain at any stage, regardless of where it is, they will be advised to stop the tests to prevent any harm. Cautiously, a nurse (Eugênia Cruz Santos – COREN 563040-ENF) will provide first aid with the appropriate materials, and an emergency vehicle will be on standby (before and after the running test) in case the participant needs to be transported to an urgent care or emergency facility.

7. BENEFITS TO THE PARTICIPANT

After the study concludes, a lecture will be held to present the results obtained, and flyers will be distributed to inform the public about the study. These actions aim to improve the sport related to the study's theme, as well as promote the practice of the sport and provide information on what is suggested regarding strength training in road running performance. Additionally, each participant will be entitled to their physical test results and their anthropometric assessment.

8. PARTICIPANT'S FREEDOM

The participant is guaranteed the freedom to respond only to questions and information that do not cause them discomfort. They also have the right to refuse to participate in the research or withdraw their consent at any stage of the study, without any penalty.

9. RESEARCH CONFIDENTIALITY

The results obtained during this study will be kept confidential, as well as the images and testimonials collected. Participants' data will be anonymized by replacing their names with numbers during data analysis. Subsequently, the study results will be published in scientific publications.

10. STATISTICAL TREATMENT

To verify the distribution of the sample, when necessary, normality will be checked using the Shapiro-Wilk or Kolmogorov-Smirnov test, taking into account the sample size. Homogeneity will be assessed through Levene's test. Differences between types of training will be examined using repeated measures ANOVA (factors: time and session) or its non-parametric equivalent (Friedman test) in case of normality rejection. For all statistical and graphical analyses, the Statistical Package for Social Sciences (SPSS), version 20®, and GraphPad Prism version 7.00 will be used, respectively. A significance level of $p<0.05$ will be adopted.

11. OUTCOMES

11.1. Primary

- Running performance in a 3000-meter time trial race among street runners subjected to two types of stress-related time protocols associated with concentric/eccentric muscle actions versus isometric in the TF.

11.2. Secondary

- Pre-test peak torque against the 3000-meter street runners subjected to two types of time protocols under tension associated with concentric/eccentric muscle actions versus isometric in the TF.
- Rating perception of effort (RPE) after a 3000-meter road running time trial under two types of time protocols under tension, associated with concentric/eccentric muscle actions versus isometric in the TF.
- Ground contact time (GCT) after a 3000-meter road runners' time trial against a clock, subjected to two types of tension protocols associated with concentric/eccentric muscle actions versus isometric in the TF.

- Levels of neuromuscular fatigue (CMJ) after a 3000-meter time trial race among road runners subjected to two types of time-under-tension protocols associated with isometric and eccentric muscle actions in the TF.

12. REFERENCES

ADAMS, Douglas et al. Validity and reliability of a commercial fitness watch for measuring running dynamics. **Journal of Orthopaedic & Sports Physical Therapy**, v. 46, n. 6, p. 471-476, 2016.

ANDERSON, Tim. Biomechanics and running economy. **Sports medicine**, v. 22, p. 76-89, 1996.

ARAMPATZIS, Adamantios et al. Mechanical properties of the triceps surae tendon and aponeurosis in relation to intensity of sport activity. **Journal of biomechanics**, v. 40, n. 9, p. 1946-1952, 2007.

BENTO, Paulo Cesar Barauce et al. Peak torque and rate of torque development in elderly with and without fall history. **Clinical biomechanics**, v. 25, n. 5, p. 450-454, 2010.

BLAGROVE, Richard C. et al. Strength and conditioning for adolescent endurance runners. **Strength & Conditioning Journal**, v. 42, n. 1, p. 2-11, 2020.

BLAGROVE, Richard C.; HOWATSON, Glyn; HAYES, Philip R. Use of loaded conditioning activities to potentiate middle-and long-distance performance: a narrative review and practical applications. **The Journal of Strength & Conditioning Research**, v. 33, n. 8, p. 2288-2297, 2019.

BOGDANIS, Gregory C. et al. Effects of low volume isometric leg press complex training at two knee angles on force-angle relationship and rate of force development. **European journal of sport science**, v. 19, n. 3, p. 345-353, 2019.

BORG, Gunnar A. Psychophysical bases of perceived exertion. **Medicine and science in sports and exercise**, v. 14, n. 5, p. 377-381, 1982.

CHANG, Wei-Ling; SHIH, Yi-Fen; CHEN, Wen-Yin. Running injuries and associated factors in participants of ING Taipei Marathon. **Physical Therapy in Sport**, v. 13, n. 3, p. 170-174, 2012.

DAMASCENO, Mayara V. et al. Effects of resistance training on neuromuscular characteristics and pacing during 10-km running time trial. **European journal of applied physiology**, v. 115, p. 1513-1522, 2015.

DAVIES, Timothy B. et al. Effect of movement velocity during resistance training on dynamic muscular strength: a systematic review and meta-analysis. **Sports medicine**, v. 47, p. 1603-1617, 2017.

DOMA, Kenji; DEAKIN, Glen Bede. The acute effects intensity and volume of strength training on running performance. **European journal of sport science**, v. 14, n. 2, p. 107-115, 2014.

FERRAUTI, Alexander; BERGERMANN, Matthias; FERNANDEZ-FERNANDEZ, Jaime. Effects of a concurrent strength and endurance training on running performance and running

economy in recreational marathon runners. **The Journal of Strength & Conditioning Research**, v. 24, n. 10, p. 2770-2778, 2010.

FESTA, Luca et al. Effects of flywheel strength training on the running economy of recreational endurance runners. **The Journal of Strength & Conditioning Research**, v. 33, n. 3, p. 684-690, 2019.

FLETCHER, Jared R.; ESAU, Shane P.; MACINTOSH, Brian R. Changes in tendon stiffness and running economy in highly trained distance runners. **European journal of applied physiology**, v. 110, n. 5, p. 1037-1046, 2010.

FOSTER, Carl et al. A new approach to monitoring exercise training. **The Journal of Strength & Conditioning Research**, v. 15, n. 1, p. 109-115, 2001.

GÄBLER, Martijn et al. The effects of concurrent strength and endurance training on physical fitness and athletic performance in youth: a systematic review and meta-analysis. **Frontiers in physiology**, v. 9, p. 336232, 2018.

GAUFFIN, Håkan et al. Maintaining motivation and health among recreational runners: Panel study of factors associated with self-rated performance outcomes at competitions. **Journal of Science and Medicine in Sport**, v. 22, n. 12, p. 1319-1323, 2019.

GENTIL, P.; OLIVEIRA, E.; BOTTARO, M. Time under Tension and Blood Lactate Response during Four Different Resistance Training Methods. **Journal of Physiological Anthropology**, v. 25, n. 5, p. 339–344, 2006.

HAWLEY, John A. et al. Integrative biology of exercise. **Cell**, v. 159, n. 4, p. 738-749, 2014.
JACKSON, Andrew S.; POLLOCK, Michael L. Generalized equations for predicting body density of men. **British journal of nutrition**, v. 40, n. 3, p. 497-504, 1978.

JACKSON, Andrew S.; POLLOCK, Michael L. Generalized equations for predicting body density of men. **British journal of nutrition**, v. 40, n. 3, p. 497-504, 1978.

JOHNSON, Ronald E. et al. Strength training in female distance runners: impact on running economy. **The Journal of Strength & Conditioning Research**, v. 11, n. 4, p. 224-229, 1997.

KNIHS, Débora Aparecida et al. Reliability and sensitivity of countermovement jump-derived variables in detecting different fatigue levels. **Journal of Physical Education**, v. 32, p. e3232, 2022.

KUBO, Keitaro et al. Time course of changes in the human Achilles tendon properties and metabolism during training and detraining in vivo. **European journal of applied physiology**, v. 112, p. 2679-2691, 2012.

LEE, Duck-chul et al. Running as a key lifestyle medicine for longevity. **Progress in cardiovascular diseases**, v. 60, n. 1, p. 45-55, 2017.

LI, Fei et al. Effects of complex training versus heavy resistance training on neuromuscular adaptation, running economy and 5-km performance in well-trained distance runners. **PeerJ**, v. 7, p. e6787, 2019.

LICHTWARK, Glen A.; BOUGOULIAS, K.; WILSON, Alan M. Muscle fascicle and series elastic element length changes along the length of the human gastrocnemius during walking and running. **Journal of biomechanics**, v. 40, n. 1, p. 157-164, 2007.

Lorimer A., Wader M., Pearson S. Validation of Contact Time, Step Rate, and Vertical Oscillation as Determined by the Garmin HRM-Run System. **High Performance Sport New Zealand**; Auckland, New Zealand: 2016.

LUCKIN-BALDWIN, Kate M. et al. Strength training improves exercise economy in triathletes during a simulated triathlon. **International journal of sports physiology and performance**, v. 16, n. 5, p. 663-673, 2021.

LUM, Danny et al. Effects of isometric strength and plyometric training on running performance: a randomized controlled study. **Research quarterly for exercise and sport**, v. 94, n. 1, p. 263-271, 2023.

MACINNIS, Martin J.; GIBALA, Martin J. Physiological adaptations to interval training and the role of exercise intensity. **The Journal of physiology**, v. 595, n. 9, p. 2915-2930, 2017.

MANG, Zachary Aaron et al. Aerobic adaptations to resistance training: the role of time under tension. **International journal of sports medicine**, v. 43, n. 10, p. 829-839, 2022.

MIDGLEY, Adrian W.; MCNAUGHTON, Lars R.; JONES, Andrew M. Training to enhance the physiological determinants of long-distance running performance: can valid recommendations be given to runners and coaches based on current scientific knowledge?. **Sports medicine**, v. 37, p. 857-880, 2007.

MIKKOLA, Jussi et al. Effect of resistance training regimens on treadmill running and neuromuscular performance in recreational endurance runners. **Journal of sports sciences**, v. 29, n. 13, p. 1359-1371, 2011.

MOOSES, Martin et al. Shorter ground contact time and better running economy: evidence from female Kenyan runners. **The Journal of Strength & Conditioning Research**, v. 35, n. 2, p. 481-486, 2021.

MOREIRA, Alexandre et al. Validez y reproducibilidad de receptores GPS en relación de la distancia recorrida. **Revista Andaluza de Medicina del Deporte**, v. 6, n. 4, p. 146-150, 2013.

NOAKES, Timothy D.; MYBURGH, Kathryn H.; SCHALL, Robert. Peak treadmill running velocity during the V O2 max test predicts running performance. **Journal of sports sciences**, v. 8, n. 1, p. 35-45, 1990.

PAAVOLAINEN, Leena et al. Explosive-strength training improves 5-km running time by improving running economy and muscle power. **Journal of applied physiology**, 1999.

PRIETO-GONZÁLEZ, Pablo; SEDLACEK, Jaromir. Effects of running-specific strength training, endurance training, and concurrent training on recreational endurance athletes' performance and selected anthropometric parameters. **International journal of environmental research and public health**, v. 19, n. 17, p. 10773, 2022.

RØNNESTAD, Bent R.; MUJIKA, Iñigo. Optimizing strength training for running and cycling endurance performance: A review. **Scandinavian journal of medicine & science in sports**, v. 24, n. 4, p. 603-612, 2014.

SANT'ANA, Leandro; BARA-FILHO, Maurício Gáttas; VIANNA, Jeferson Macedo. Monitoramento da carga de treinamento na corrida: Aspectos fisiológicos e metodológicos na aplicabilidade prática desta modalidade: Monitoramento da Carga de treinamento na

Corrida. **Research, Society and Development**, v. 10, n. 9, p. e23110916986-e23110916986, 2021.

SEDANO, Silvia et al. Concurrent training in elite male runners: the influence of strength versus muscular endurance training on performance outcomes. **The Journal of Strength & Conditioning Research**, v. 27, n. 9, p. 2433-2443, 2013.

SUNDBY, Øyvind H.; GORELICK, Mark LS. Relationship between functional hamstring: quadriceps ratios and running economy in highly trained and recreational female runners. **The Journal of Strength & Conditioning Research**, v. 28, n. 8, p. 2214-2227, 2014.

TAIPALE, R. S. et al. Strength training in endurance runners. **International journal of sports medicine**, p. 468-476, 2010.

TANIMOTO, Michiya; ISHII, Naokata. Effects of low-intensity resistance exercise with slow movement and tonic force generation on muscular function in young men. **Journal of applied physiology**, v. 100, n. 4, p. 1150-1157, 2006.

TEIXEIRA, Pedro J. et al. Exercise, physical activity, and self-determination theory: a systematic review. **International journal of behavioral nutrition and physical activity**, v. 9, p. 1-30, 2012.

TOZEI, William Peneda et al. Método tradicional vs. série pareada agonista-antagonista para membros inferiores: há diferenças no volume total e repetições máximas?. **Corpoconsciência**, p. 134-148, 2021.

YAMAGUCHI, Taichi; TAKIZAWA, Kazuki; SHIBATA, Keisuke. Acute effect of dynamic stretching on endurance running performance in well-trained male runners. **The Journal of Strength & Conditioning Research**, v. 29, n. 11, p. 3045-3052, 2015.

ZOURDOS, Michael C. et al. Effects of dynamic stretching on energy cost and running endurance performance in trained male runners. **The Journal of Strength & Conditioning Research**, v. 26, n. 2, p. 335-341, 2012.
